# Malignant perivascular epithelioid cell tumor (PEComa) of the uterus with late renal and pulmonary metastases: a case report with review of the literature

**DOI:** 10.1186/1746-1596-2-45

**Published:** 2007-12-03

**Authors:** Henry B Armah, Anil V Parwani

**Affiliations:** 1 Department of Pathology, University of Pittsburgh Medical Center, Pittsburgh, PA, USA

## Abstract

**Background:**

Perivascular epithelioid cell tumor (PEComa), other than angiomyolipoma (AML), clear cell sugar tumor (CCST), and lymphangioleiomyomatosis (LAM), is a very rare mesenchymal tumor with an unpredictable natural history. The uterus is the most prevalent reported site of involvement of PEComa-not otherwise specified (PEComa-NOS). To the best of our knowledge, about 100 PEComa-NOS have been reported in the English Language medical literature, of which 38 were uterine PEComa-NOS. These reported cases of uterine PEComa-NOS have usually shown clinically benign behavior, but 13 tumors, three of them associated with tuberous sclerosis complex (TSC), exhibited local aggressive behavior and four of them showed distant metastases.

**Case presentation:**

We report the case of a 59-year-old woman, who presented with renal and pulmonary lesions seven years after the initial diagnosis of uterine leiomyosarcoma. Left nephrectomy and right middle lobe wedge resection were performed. Histological and immunohistochemical analysis of the renal and pulmonary lesions, in addition to retrospective re-evaluation of the previous uterine tumor, led to the final diagnosis of malignant uterine PEComa with late renal and pulmonary metastases. All three lesions had the typical histological appearance of PEComa-NOS showing a biphasic growth pattern with continuous transition between spindle cells and epithelioid cells, often arranged around vascular spaces. Immunohistochemically, the tumor cells of both phenotypes in all three lesions stained for melanocytic (HMB-45 and Melan-A/MART-1) and myoid (desmin, smooth muscle actin, and muscle-specific actin/all muscle actin/HHF-35) markers.

**Conclusion:**

The findings indicate that despite the small number of reported cases, PEComas-NOS should be considered tumors of uncertain malignant potential, and metastases to other organs might become evident even several years after the primary diagnosis.

## Background

The World Health Organization defines perivascular epithelioid cell tumors (PEComas) as "mesenchymal tumors composed of histologically and immunohistochemically distinctive perivascular epithelioid cells (PECs)" [1]. In 1991, Pea and colleagues [2] first noted this unusual cell in both angiomyolipoma (AML) and clear cell sugar tumor (CCST) of the lung. One year later, Bonetti and colleagues [3] proposed a cellular link between AML, CCST, and lymphangioleiomyomatosis (LAM), their association with tuberous sclerosis complex (TSC), and advanced the concept of a family of neoplasms composed of this distinctive cell which was "immunoreactive with melanocytic markers, and exhibit an epithelioid appearance, a clear-acidophilic cytoplasm, and a perivascular distribution". In 1996, Zamboni et al [4] reported the first case of pancreatic CCST and suggested the name PEComa for these neoplasms composed of a pure proliferation of PECs. There is no known normal cellular counterpart to this PEC, and a precursor lesion for PEComas has not been described [1]. Subsequently, the PEComa family of tumors has grown to include AML, CCST, LAM, and a number of rare unusual visceral, intraabdominal, soft tissue and bone tumors, which have been described under a variety of names, including "clear cell myomelanocytic tumor (CCMMT) of the falciform ligament/ligamentum teres", "abdominopelvic sarcoma of perivascular epithelioid cells", and "primary extrapulmonary clear cell sugar tumor", among others. This latter group of rare, morphologically and immunophenotypically similar tumors arising at a variety of visceral (commonly gastrointestinal, gynecological and genitourinary) and soft tissue (commonly retroperitoneal, abdominopelvic and cutaneous) sites have been collectively termed non-AML, non-LAM, non-CCST PEComas; or PEComas other than AML, LAM or CCST; or PEComas-not otherwise specified (PEComas-NOS) [1]. Generally, CCMMT is now not considered a distinct entity, but rather fall within the morphological spectrum of PEComas-NOS [1].

To the best of our knowledge, about 100 PEComas-NOS have been reported in the English Language medical literature, of which 38 were uterine PEComas-NOS [1,5-17]. These 38 reported cases of uterine PEComas-NOS have usually shown clinically benign behavior, but thirteen tumors exhibited locally aggressive behavior [5-9]. Three of these thirteen locally aggressive uterine PEComa-NOS were associated with TSC [5,6], whilst four of them showed distant metastases to liver, lungs, intestines, bone and lymph nodes up to seven years after resection of the uterine tumors [5,7,9]. Although there is a strong association between TSC, AML, LAM and CCST; this association is much less clear for the rarer PEComas-NOS [1]. Since relatively few cases of malignant PEComa have been reported and the duration of follow-up relatively short in the reported cases of PEComa, firm criteria for malignancy have yet to be established. However, a recent review report suggested criteria for malignancy including a size of >8.0 cm, mitotic count of >1 per 50 high-power fields (HPFs) and necrosis; and these three criteria helped to stratify PEComas into benign, uncertain malignant potential, and malignant [8]. Unfortunately, until more cases of this rare tumor are evaluated in a systematic fashion, firm criteria for malignancy remain uncertain. We report herein the case of a 59-year-old woman who presented with renal and pulmonary lesions seven years after the initial diagnosis of uterine leiomyosarcoma, but histological and immunohistochemical analysis of the renal, pulmonary and previous uterine lesions led to the final diagnosis of malignant uterine PEComa-NOS with late renal and pulmonary metastases.

## Case presentation

A 59-year-old woman with a past medical history of hysterectomy and bilateral salpingo-ophrectomy for uterine leiomyosarcoma (American Joint Commission on Cancer Staging; AJCC T1c N0 M0; diagnosed at an outside institution) seven years prior presented to our institution for her regular periodic computed tomography (CT) scans of the abdomen, chest and pelvis at 6-month intervals as tumor follow-up investigations. These CT scans revealed several coalescing rim-enhancing nodules (altogether measuring 6.0 cm) in the upper and middle poles of her left kidney and a solitary rim-enhancing nodule (measuring 1.5 cm) in her right middle lobe of lung, which were absent on her previous CT scans six months earlier. The clinical impression was metastasis from her previous uterine leiomyosarcoma. No additional nodules were identified in the brain, gastrointestinal (GI) tract, liver, spleen, pancreas, bladder and bones on subsequent staging magnetic resonance imaging (MRI) of the brain, abdomen, chest and pelvis, and positron emission tomography (PET) scan. The patient did not have any stigmata or family history of TSC, and had no history of melanoma. General physical examination was unremarkable. Her hemogram, urine and blood biochemical analyses were within normal ranges.

**Figure 1:**
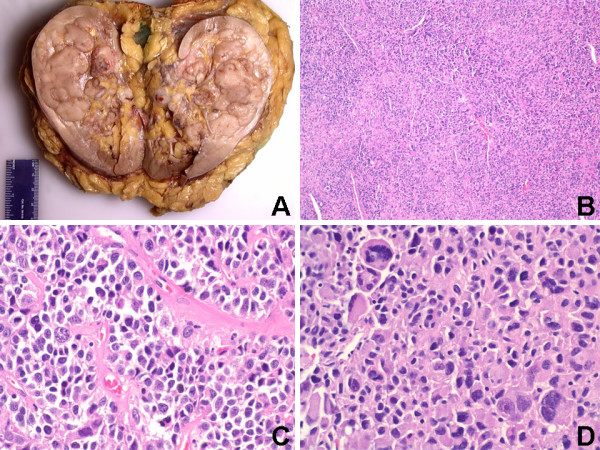
(A) Sharply demarcated gray-tan nodular tumor occupying the upper and middle poles with focally hemorrhagic areas on cut surface (Gross). (B) Biphasic renal tumor composed mainly of medium to large epithelioid cells and focal areas of spindle cells around numerous small blood vessels (H&E, Original magnification ×100). (C) Perivascular epithelioid cells arranged in solid nests or cords with well-defined cytoplasmic borders and abundant cytoplasm that varied from eosinophilic and granular to clear (H&E, Original magnification ×400). (D) Moderate-to-severe nuclei pleomorphism and hyperchromatism with bizarre multinucleated giant cells and mitoses (H&E, Original magnification ×400).

A left radical nephrectomy and video-assisted thoracoscopic surgical wedge resection of the right middle lobe solitary nodule were performed. Grossly, her resected left kidney revealed a sharply demarcated and lobulated firm gray-tan nodular tumor (7.5 × 6.2 × 5.4 cm) occupying the upper and middle poles, and showed focally hemorrhagic areas on cut surface (Figure 1A). The interface between tumor and uninvolved kidney was sharp (Figure 1A). A 3.0 cm tan tumor thrombus protruded from the renal vein resection margin, whilst the renal artery and ureter resection margins were grossly free of tumor. The tumor grossly infiltrated into the renal sinus and extended broadly to, but not through, both the anterior and posterior renal capsules. Gross infiltration of tumor into the perinephric adipose tissue was absent. Her wedge-resected right middle lobe of lung revealed a sharply demarcated firm gray-tan nodule (2.3 × 2.2 × 2.0 cm) with focally hemorrhagic overlying pleura. The cut surface of the tumor was focally hemorrhagic. The tumor extends to, but not through the overlying pleura, and was at least 0.5 cm from the nearest stapled resection margin.

Histologically, the renal tumor was biphasic and composed mainly of medium to large epithelioid cells and focal areas of spindle/elongated cells around numerous small blood vessels (Figures 1B & 1C). The medium to large epithelioid neoplastic cells were arranged in solid nests or cords, and had well-defined cytoplasmic borders with abundant cytoplasm that varied from eosinophilic and granular to clear (Figure 1C). The nuclei of the epithelioid cells were mainly round, centrally located, and vesicular, with moderate-to-severe pleomorphism and hyperchromatism (Figures 1C &1D). Extensive cellular atypia, including bizarre multinucleated giant cells with large nuclei and nucleoli, was present (Figure 1D). Conspicuous nucleoli and mitoses were frequently present (Figure 1D). Mitotic count for the renal tumor was high (60 per 50 HPFs). There was microscopic evidence of tumor invasion of the renal sinus and the renal vein, and extensive neoplastic cell invasion of smaller veins. The adrenal gland was unremarkable and was not invaded by tumor, and the non-neoplastic kidney revealed mild nephrosclerosis.

Microscopic examination of the pulmonary tumor (not shown) exhibited the same morphological features as the renal tumor described above (Figures 1B–1D). The interface between tumor and uninvolved lung was sharp, and several foci of hemorrhage and necrosis were present. Mitotic count for the pulmonary tumor was high (45 per 50 HPFs). The non-neoplastic lung tissue was unremarkable (not shown). Based on her history of status post hysterectomy for uterine leiomyosarcoma (diagnosed at an outside institution) seven years prior and the biphasic histologic appearance of spindle and epithelioid cells (with clear cell areas) with prominent perivascular distribution of neoplastic cells in both the renal and pulmonary tumors, a diagnosis of malignant uterine PEComa with renal and pulmonary metastases was considered. The pathology report, original hematoxylin-eosin (H&E) slides, and formalin-fixed paraffin embedded blocks of the prior uterine tumor were requested from the outside institution. The original H&E slides and new H&E slides re-cut from the paraffin blocks were evaluated, and sections from all three lesions (uterine, renal and pulmonary) were concurrently subjected to immunohistochemical analysis. The pathology report from the outside institution indicated that the prior uterine tumor diagnosed as uterine leiomyosarcoma measured 6.0 cm in its greatest dimension, with a pathologic TMN staging of pT1c N0 M0. Microscopic examination of the previous uterine tumor (not shown) exhibited the same morphological features as both the pulmonary (not shown) and renal (Figures 1B–1D) tumors described above.

**Figure 2:**
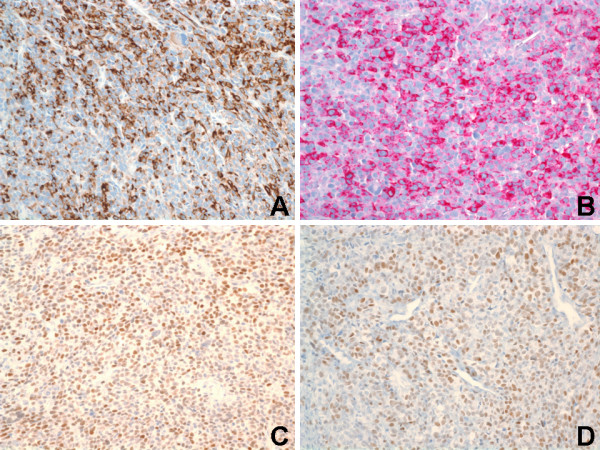
Immunohistochemical staining of renal tumor. (A) Both epithelioid and spindle cells were strong and diffusely positive for desmin (Immunoperoxidase, Original magnification ×200). (B) Both epithelioid and spindle cells were strong and diffusely positive for HMB-45 (Immunoperoxidase, Original magnification ×200). (C) Estrogen receptor (ER) immunostaining showed strong and diffuse nuclear reactivity in tumor cells (Immunoperoxidase, Original magnification ×200). (D) Progesterone receptor (PR) immunostaining showed strong and moderate nuclear reactivity in tumor cells (Immunoperoxidase, Original magnification ×200).

**Figure 3:**
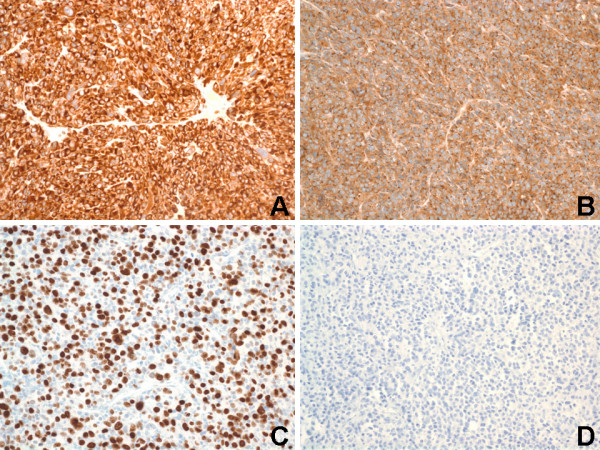
Immunohistochemical staining of renal tumor. (A) Tumor cells showed strong and diffusely reactivity for BCL-2 (Immunoperoxidase, Original magnification ×200). (B) Tumor cells showed strong and diffusely reactivity for CD99 (Immunoperoxidase, Original magnification ×200). (C) Ki67 immunostaining showed a proliferative index of 70% (Immunoperoxidase, Original magnification ×200). (D) Tumor cells showed negative reactivity for renal cell carcinoma marker (RCC) (Immunoperoxidase, Original magnification ×200).

Immunohistochemically, both the epithelioid and spindle neoplastic cells of the renal tumor were strong and diffusely (>80% of the neoplastic cells) positive for desmin (cytoplasmic and membranous) {Ventana, Tucson, Ariz} [Figure 2A], HMB-45 (cytoplasmic) {Ventana} [Figure 2B], Melan-A/MART-1 (cytoplasmic) {BioCare Medical, Walnut Creek, CA}, smooth muscle actin (cytoplasmic and membranous) {Dako, Carpinteria, CA}, muscle-specific actin/all muscle actin/HHF-35 (cytoplasmic and membranous) {Dako} [not shown]. The estrogen receptor (ER) {Ventana} [Figure 2C] and progesterone receptor (PR) {Ventana} [Figure 2D] immunostains showed strong nuclear reactivity in 60–80% and 30–50% of tumor cells, respectively. The renal tumor cells were strong and diffusely (>80% of the neoplastic cells) positive for BCL-2 (cytoplasmic and membranous) {Ventana} [Figure 3A], CD99 (membranous) {Ventana} [Figure 3B], and cyclin D1 (nuclear) {Ventana} [not shown]. A proliferative index of 70% was noted with Ki67 immunostaining (nuclear) {Ventana} [Figure 3C]. The tumor cells showed focal (<20% of the neoplastic cells), but strong, immunoreactivity for c-kit/CD117 (membranous) {Ventana}, vimentin (cytoplasmic and membranous) {Ventana}, epithelial membrane antigen (EMA) {cytoplasmic and membranous} [Ventana], and CD10 (membranous) {Ventana} [not shown]. The renal neoplastic cells failed to stain with antibodies against renal cell carcinoma marker (RCC) {Ventana} [Figure 3D], S-100 protein (Ventana), chromogranin (Ventana), cytokeratins (AE1/AE3, 8/18, 7/20) {Ventana}, CD34 (Ventana), and myogenin (Ventana) {not shown}. The immunoprofile of the pulmonary (not shown) and uterine (not shown) tumors were identical to that of the renal tumor described above (Figures 2A–2D & Figures 3A–3D). For all the three tumors in general, staining for the melanocytic markers (HMB-45 and Melan-A) were more intense in the epithelioid cells compared to the spindle cells. Staining for the myoid markers (desmin, smooth muscle actin, and muscle-specific actin) were variable in the cytoplasm and more prominent adjacent to the cytoplasmic membrane, and more intense in the spindle cells compared to the epithelioid cells. Positive controls were used for all markers as follows: HMB-45 and Melan-A – melanoma; RCC – renal cell carcinoma; S100 – schwannoma; desmin, smooth muscle actin, muscle-specific actin, and myogenin – GI smooth muscle; CD117 – GI mast cells and Cajal cells; cytokeratins – GI epithelial tissue; vimentin – GI mesenchymal tissue; and CD34 – tonsillar endothelial tissue. Slides stained omitting the primary antibody were used as negative controls.

On the basis of the above histopathological and immunohistochemical features, a definitive diagnosis of malignant uterine PEComa with late renal and pulmonary metastases was rendered. The patient elected not to have any adjuvant chemotherapy or radiotherapy. Five follow-up CT scans of the brain, abdomen, chest and pelvis, and PET scans performed at 3-month intervals after surgery revealed no nodules. She is alive with no evidence of local recurrence or distant metastasis after 15 months of follow-up, and scheduled to have regular periodic CT scans of the abdomen, chest and pelvis at 3-month intervals as her tumor follow-up plan.

## Discussion

In this report, we describe a case of unusual epithelioid tumors in the kidney and the lung seven years after the initial diagnosis of uterine leiomyosarcoma, with pathological and immunohistochemical features entirely compatible with those of a PEComa. PEComas are characterized by epithelioid to spindle cells with eosinophilic to clear cytoplasm, an intimate relationship with blood vessels, and demonstrate positive immunostaining for markers of both melanocytic (HMB45, Melan-A, tyrosinase, microphthalmia transcription factor) and myoid (desmin, smooth muscle actin, muscle-specific actin, caldesmon, calponin) differentiation [1]. PEComas-NOS have now been reported in almost every body site and the growing list of reported sites include gynecological, genitourinary, gastrointestinal, extremities and the skin, as well as single reports in the heart, breast, oral cavity, orbit, and skull base [1,5-17]. The uterus is the most prevalent reported site of involvement of PEComa-NOS (accounting for 38 out of about 100 reported cases). The other common sites are the gastrointestinal tract, genitourinary tract and retroperitoneum, whilst rare sites include somatic soft tissue, skin and bone. Almost all the reported non-uterine PEComas-NOS have been in women [1,5-17]. The vast majority of PEComas-NOS have been described in females (as in the case herein presented) [1,5-17], and therefore hormones may play a role in their pathogenesis and/or phenotypic cellular manifestations. Estrogen receptor (ER) and progesterone receptor (PR) expression, that have been reported primarily in the spindle cell component of PEComas [5,10], may play a role in the development of this morphologic pattern. The case herein presented showed moderate-to-strong positive nuclear staining for both ER and PR in both the spindle and epithelioid cell components of all three lesions (uterine, renal and pulmonary), although the ER staining was always more intense than PR staining.

PEComas are of interest primarily because of their immunoreactivity with melanocytic and myoid markers. They are also almost always negative for S-100 protein and cytokeratins. Folpe and colleagues [8] recently reviewed all reported cases of PEComa up to 2005 (61 cases). In their review, 100% were HMB-45 positive, 59% were smooth muscle actin positive, 41% were Melan-A positive, 33% were CD117 positive, 31% were desmin positive, 11% were S-100 positive, and 0% was cytokeratin positive. Therefore, nearly all PEComas are immunoreactive for HMB-45 and/or Melan-A, and many are positive for smooth muscle actin, whereas desmin staining appears to be somewhat less common. Elongated spindle cells in PEComas are characterized by prominent smooth muscle-specific filaments, while the epithelioid component does not usually contain high numbers of such filaments [8]. The case herein presented showed strong and diffuse staining for both HMB-45 and MelanA in both the spindle and epithelioid cell components of all three lesions (uterine, renal and pulmonary), with more intense staining in the epithelioid cells compared to the spindle cells. In the present case, staining for muscle-specific actin, smooth muscle actin, and desmin was more evident in the area adjacent to the cytoplasmic membrane, with more intense staining in the spindle cells compared to the epithelioid cells.

Not unexpectedly, the uterine tumor of the case herein presented was initially misdiagnosed as a uterine leiomyosarcoma at an outside institution seven years prior. Myomelanocytic marker expression was found to be prominent in both components of all the three lesions in the case herein presented. Controversy exists regarding the minimum criteria for the diagnosis of malignant PEComa [18,19]. In studies of uterine tumors demonstrating epithelioid morphology, clear cell areas and HMB-45 positivity, these authors [18,19] argued that these lesions represented epithelioid smooth muscle tumors with focal melanocytic differentiation and not PEComas. This view was based largely on the fact that the tumors looked like classic leiomyosarcomas with spindled and epithelioid areas, and stained with desmin. Nevertheless, these authors [18,19] still advocated performing immunohistochemistry for HMB-45 in all uterine epithelioid smooth muscle tumors, in order to identify patients who should be investigated for TSC. It is also noteworthy that only focal HMB-45 staining was seen in their cases, unlike the diffuse expression in the case herein presented and nearly all the reported cases of uterine PEComa [1,5-11]. Additionally, 31% of PEComas showed at least focal desmin staining in a recent review of all reported cases up to 2005 (61 cases) [8], and therefore desmin staining of uterine epithelioid tumors with clear cell areas and HMB-45 positivity does not exclude the diagnosis of PEComa. We are therefore of the view that tumors with diffuse myomelanocytic differentiation should be regarded as being related to the PEComa family irrespective of site of origin or desmin positivity, and that it is the characteristic morphology and immunophenotype that warrants separating these tumors from classic leiomyosarcoma that exhibit only muscle differentiation. However the clinicopathological significance of HMB-45 positivity in uterine epithelioid smooth muscle tumors is not known.

In addition to epithelioid smooth muscle tumors (epithelioid leiomyosarcoma and epithelioid leiomyoma), the other important differential diagnosis of PEComa include malignant melanoma, clear cell sarcoma of tendon and aponeuroeses (melanoma of the soft parts), alveolar soft part sarcoma, endometrial stromal sarcoma with clear cell features, uterine tumor resembling sex cord tumor, carcinoma (especially renal cell and adrenocortical carcinoma), paraganglioma, angiomyolipoma, and any other tumor with focal or prominent clear cell change. Malignant melanoma and clear cell sarcoma of tendon and aponeuroeses can be differentiated from PEComas based on S-100 positivity; however, up to 11% of PEComas express S-100 as well [8]. The additional important features for the diagnosis of PEComa in this context include negative history for melanoma, visceral location of tumor, perivascular accentuation of tumor cells, immunoreactivity for myoid markers (smooth muscle actin, muscle-specific actin, and desmin), and absence of the t(12:22) translocation. Although S-100 negative melanomas have rarely been described, tumors with S-100 negativity, strong and diffuse melanocytic marker positivity, and actin immunoreactivity should be designated as PEComas based on morphology and immunophenotype. Pitfalls in the diagnosis of PEComas include aberrant staining of cells with melanocytic markers. However, diffuse and multiple melanocytic marker expression is highly reliable for melanocytic differentiation. Focal or weak positivity can be disregarded, however, and do not warrant the diagnosis of PEComa. The case herein presented was strong and diffusely positive with at least two melanocytic markers (HMB45 and Melan-A), which is not a finding in other sarcomas. Angiomyolipoma can be ruled out because the present case lacked lipomatous elements and showed a biphasic cellular population. However, PEComa and monophasic epithelioid angiomyolipoma are probably very closely related, if not the same entity. Endometrial stromal sarcoma and uterine tumor resembling sex cord tumor can be ruled out because of the presence of prominent perivascular accentuation of tumor cells and diffuse, rather than focal, positive staining of HMB-45 in PEComa. PEComa can be distinguished from paraganglioma in that the former is negative for chromogranin A, synaptophysin, and S-100 protein, and the latter shows more organoid growth. The expression of melanocytic markers (HMB-45 and MART-1/Melan-A) and the lack of immunoreactivity for cytokeratins and RCC marker argue against the diagnosis of carcinoma.

Clinically, most PEComas follow a benign course [1]. Malignant PEComas-NOS are being increasingly reported, several originating in the uterus and others arising in the jejunum, ileum, prostate, pelvis, skull base, broad ligament and somatic soft tissue [1,5-17]. Since relatively few malignant PEComas have been reported, firm criteria for malignancy have yet to be established. However, Folpe and colleagues [8] recently suggested criteria for malignancy including a size of >8.0 cm, mitotic count of >1 per 50 high power fields (HPFs) and necrosis, with benign, uncertain malignant potential and malignant categories based on the presence of none, 1 or ≥ 2 of these three criteria, respectively. Infiltrative growth or edges, marked hypercellularity and marked nuclear pleomorphism/atypia may be secondary features suggesting aggressive behaviour or malignancy [1,8,11]. A Ki-67 labeling index of 5% of neoplastic cells has been observed in a uterine PEComa that have behaved aggressively [7]. In the case herein presented, the size of the primary uterine tumor seven years prior was <8 cm, mitotic count was >1 per 50 HPFs and necrosis was absent. Retrospectively, this uterine tumor would be categorized as of uncertain malignant potential according to the above suggested criteria. Additionally, the uterine tumor showed all the three secondary features suggestive of aggressive behaviour or malignancy. Furthermore, the Ki-67 labeling index was greater than 5% in the uterine tumor (also in the renal and pulmonary metastases), and thus the uterine tumor was destined to behave aggressively, which it did seven years later. Dimmler and colleagues have also reported late pulmonary metastases occurring seven years after the diagnosis of a case of uterine PEComa [7]. Therefore, metastatic spread of PEComas may, in some cases, be a late complication, presenting after many years. This highlights both the need for criteria that more accurately predict the behavior of PEComas and the need for long-term follow-up of patients with PEComas, as widespread metastases may present as a late complication.

Optimal treatment for PEComas is not well established at this time. Currently, surgery is the mainstay of treatment for primary PEComa at presentation as well as for local recurrences and metastases, with the aim of obtaining clear resection margins. The role of adjuvant therapy remains unclear. Metastases have been successfully managed by resection alone [7]. Primary excision is usually curative, as most PEComas are benign. However, locally advanced or metastatic disease portends a poor prognosis and treatment strategies incorporating surgery, radiotherapy and chemotherapy have been reported [20]. Given the uncertainty of PEComa tumor biology, adjuvant therapies, including chemotherapy and immunotherapy, may be considered for patients with locally advanced or metastatic PEComa.

## Conclusion

We have described a case of malignant PEComa-NOS of the uterus with late renal and pulmonary metastases seven years after hysterectomy. Malignant PEComa should be considered in the light microscopic differential diagnosis for all pleomorphic sarcomas. PEComas-NOS show a marked female predominance, are rare but anatomically ubiquitous mesenchymal tumors that are composed of nests and sheets of usually epithelioid but occasionally spindled cells with clear to granular eosinophilic cytoplasm and a focal association with vascular spaces. They usually show immunoreactivity for both melanocytic (HMB-45 and/or Melan-A/Mart-1) and myoid/muscle-specific (actin and/or desmin) markers, which are useful for confirming the diagnosis. The mainstay of treatment is wide excision. Though most PEComas are benign, a subset behaves in a malignant fashion; size >8 cm, high mitotic index, presence of necrosis, marked cytological atypia, and/or infiltrative growth pattern may be associated with malignant behavior. However, since relatively few malignant PEComas have been reported, firm criteria for malignancy have yet to be established and the identity of the normal PEC remains elusive. Further studies on additional cases with longer clinical follow-up would be necessary to accurately predict the biologic behavior of these distinctive tumors. Finally, because PEComas can behave in an aggressive manner, careful follow up is warranted.

## Abbreviations

AML, angiomyolipoma;

CCMMT, clear cell myomelanocytic tumour;

CCST, clear cell sugar tumour;

LAM, lymphangiomyomatosis;

PEC, perivascular epithelioid cell;

PEComa, perivascular epithelioid cell tumor;

TSC, tuberous sclerosis complex

## Competing interests

The author(s) declare that they have no competing interests.

## Authors' contributions

**HBA **participated in the histopathological evaluation, performed the literature review, acquired the photomicrographs and drafted the manuscript. **AVP **conceived and designed the study, gave the final histopathological diagnosis and revised the manuscript for important intellectual content. Both authors read and approved the final manuscript.
